# Mechanisms of Immune Evasion and Immune Modulation by Lymphoma Cells

**DOI:** 10.3389/fonc.2018.00054

**Published:** 2018-03-07

**Authors:** Thomas Menter, Alexandar Tzankov

**Affiliations:** ^1^Institute of Pathology and Medical Genetics, University Hospital of Basel, Basel, Switzerland

**Keywords:** CD58, CD70, Epstein–Barr virus, HLA-G, lymphoma, microenvironment, PDL1, PD1

## Abstract

**Purpose:**

Targeting cancer cells by modulating the immune system has become an important new therapeutic option in many different malignancies. Inhibition of CTLA4/B7 and PD1/PDL1 signaling is now also being investigated and already successfully applied to various hematologic malignancies.

**Methods:**

A literature review of PubMed and results of our own studies were compiled in order to give a comprehensive overview on this topic.

**Results:**

We elucidate the pathophysiological role of immunosuppressive networks in lymphomas, ranging from changes in the cellular microenvironment composition to distinct signaling pathways such as PD1/PDL1 or CTLA4/B7/CD28. The prototypical example of a lymphoma manipulating and thereby silencing the immune system is Hodgkin lymphoma. Also other lymphomas, e.g., primary mediastinal B-cell lymphoma and some Epstein–Barr virus (EBV)-driven malignancies, use analogous survival strategies, while diffuse large B-cell lymphoma of the activated B-cell type, follicular lymphoma and angioimmunoblastic T-cell lymphoma to name a few, exert further immune escape strategies each. These insights have already led to new treatment opportunities and results of the most important clinical trials based on this concept are briefly summarized. Immune checkpoint inhibition might also have severe side effects; the mechanisms of the rather un(der)recognized hematological side effects of this treatment approach are discussed.

**Conclusion:**

Silencing the host’s immune system is an important feature of various lymphomas. Achieving a better understanding of distinct pathways of interactions between lymphomas and different immunological microenvironment compounds yields substantial potential for new treatment concepts.

## Introduction

Next to surgery, chemotherapy and radiotherapy, immunotherapy has become a new effective strategy to treat human cancer (1). This field spans from cytokine therapy, tumor vaccines, and infusions of primed T-cells to drugs specifically targeting immune checkpoint signaling such as programmed cell death 1 (PD1/CD279) and its ligand PDL1 and the cytotoxic T-lymphocyte-associated protein 4 (CTLA4/CD152) and its ligand B7, both ligands being expressed on target- or antigen-presenting cells to inhibit T-cell activation. Though initially these treatments were designed for solid cancers, this concept is now readily applied in a variety of hematolymphoid neoplasms. In addition, in hematolymphoid neoplasms, another form of “immunotherapy,” allogenic hematopoietic stem cell transplantation has been used for a long time already showing a tremendous improvement of patients’ prognosis (2).

The history of immunotherapy reaches back more than 100 years to studies of Paul Ehrlich, and, despite obvious efficacy, its application regarding the type of treatment and its targets is still controversially discussed (3). It has been studied in various animal models with inconclusive results. While immune-deficient nude mice, which display a markedly reduced amount of T-cells, do not show an increased rate of tumors (4), specifically genetically modified mice with knock-outs of *recombination activating gene 2, signal transducer and activator of transcription 1 (STAT1);* or the *gamma-interferon receptor* show increased cancer rates even if not treated with carcinogens or crossed with animals with a cancer development stimulating mutation (5, 6). The reason for the lower tumor rates in nude mice is explained by a reduced, yet sustained amount of non-thymic T-cells as well as an upregulation of innate immunity. Looking at humans, patients with iatrogenic, viral or genetically caused immunodeficiency are known to have higher rates of both, virus-related cancers, such as lymphomas, squamous cell skin cancer or Kaposi sarcomas, and of non-virus-related cancers, such as colon and lung cancer. Mechanistically, immunosurveillance of tumors, especially those, which have escaped cellular senescence (7), is mainly exerted *via* control of antigens presented by the cells *via* the major histocompatibility complex 1 (MHC1) allowing T-cells to discriminate altered, i.e., tumor cells from normal cells; CD4- and CD8-positive T-cells are the key players in controlling outgrowth of tumors (5). This mechanism puts tumor cells under pressure and leads to a selection of subclones, which have achieved the capability to evade the immune response.

In many types of tumors, cancer cells undertake considerable efforts to keep the host’s immune system at bay; this involves both the tumor cells themselves, which express immunosuppressive surface proteins such as PDL1, B7, or human leukocyte antigen (HLA) G, less MHC1 or its compound β-2 microglobulin (B2M), as well the microenvironment of the tumors, which is influenced and manipulated by the tumor cells (8). Here, upregulation of regulatory T-cell subsets and subsequent anergy of cytotoxic T-cells, crosstalk with tumor growth-promoting M2 macrophages and overexpression of the immunosuppressive enzyme indoleamine 2,3-dioxygenase (IDO) play all an important role (9–11); since the role of IDO and respective therapeutic inhibition has several times been addressed and extensively reviewed, we kindly refer to some excellent publications covering this topic (12, 13). Furthermore, both compartments secrete various factors such as interleukins and interferons as well as tumor necrosis factor alpha or transforming growth factor beta. These factors can promote tumor cell survival on the one hand and prime the microenvironment, particularly the immune system in a pro-tumorigenic manner on the other (14).

Importantly, with the broad introduction of immunotherapy it has become obvious that not all patients respond in the same way, which is both due to tumor heterogeneity (15) as well as to individual (immuno-)genetic polymorphisms (16). In order to tackle this issue, specific biomarkers are needed to allow stratification of patients to ensure tailored treatment approaches, which might increase tumor response rates.

In this review, we mainly focus on the role of lymphoma tumor cells in the immunological crosstalk and not that of the microenvironment, as this topic will be covered by the review of Dr. Xu in this journal issue.

## Hodgkin Lymphoma—The Classical Paradigm for Immunomodulative Cancer

**Figure 1:**
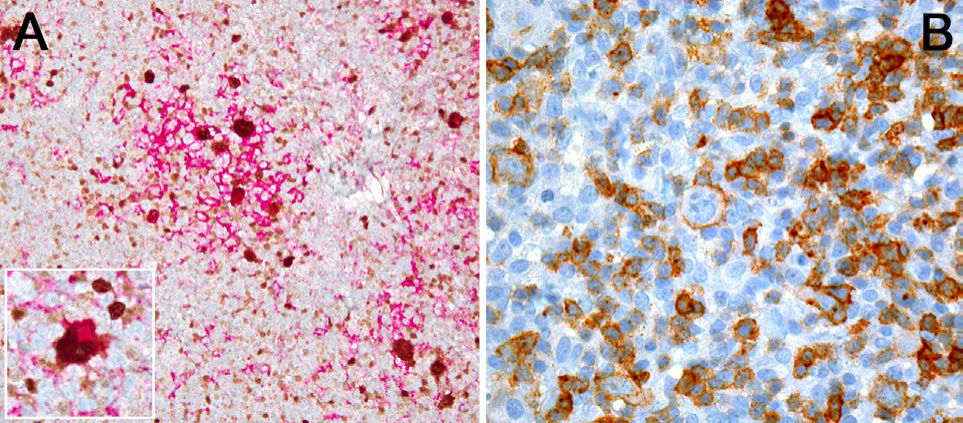
**(A)** PDL1 expression study of classical Hodgkin lymphoma with PDL1 (red chromogen)-MUM1p (brown chromogen) double-staining; note that a lot of PDL1^+^ cells, corresponding to tumor-infiltrating macrophages, do not express MUM1p while yielding dendroid cytoplasmic projections and form “immunosuppressive microniches,” in which PDL1 and MUM1p co-expressing Hodgkin- and Reed–Sternberg cells (see also inset) are scattered. **(B)** PD1 expression by single tumor cells (large ones) and plenty of tumor-infiltrating lymphocytes in T-cell- and histiocyte-rich B-cell lymphoma.

Classical Hodgkin lymphoma (cHL) comprises about 20% of lymphoid malignancies. Before the development of effective chemo- and radiotherapy regimens, it was a fatal disease (17) with patients dying—apart from mechanical problems due lymphoma burden—mainly due to infections because of severe immunosuppression caused by the cHL, exemplifying the importance of the interaction between tumor cells and the immune system. Another peculiar feature of cHL is the fact that the tumor cells [Hodgkin- and Reed–Sternberg cells (HRS cells)] comprise less than 1% of the lymphoma mass, and the majority of the tumor bulk is constituted by reactive or inflammatory cells in varying compositions, which depends on the cHL subtype. HRS cells both rely on their microenvironment on the one hand and need to specifically silence it on the other in order to prevent being attacked by it. This has been shown for T-cells as well as for tumor-associated macrophages (TAM). Regarding the latter, it has been shown that HRS cells induce PDL1 expression in macrophages (Figure 1A) in order to boost the immunosuppressive environment (18). Additionally, TAM and tumor-infiltrating lymphocytes express PD1, thus PD1/PDL1 blockade can both stop their immunosuppressive abilities and turn on tumor-surveilling attributes (19). It has been shown that the HRS cells are derived from germinal center B-cells as they carry clonally rearranged and somatically mutated immunoglobulin heavy- and light-chain genes (20, 21). HRS cells show a global downregulation of B-cell-related gene expression (22), which explains their specific immunoprofile. Genetic drivers of HRS cells are mutations in the nuclear factor kappa-light-chain enhancer of activated B cells (NF-κB) pathway, of compounds of the JAK–STAT signaling and genes involved in MHC composition and expression, and communication with T-cells (23). Deciphering the mutational landscape of HRS cells has helped to get new insights into tumorigenesis of cHL as well as elucidating mechanisms how this tumor interacts with and, thus, manipulates the immune system (24, 25).

**Figure 2:**
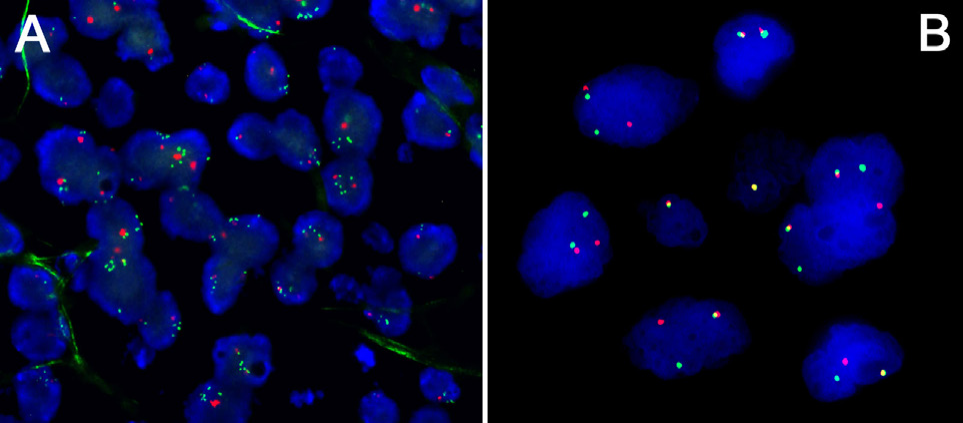
**(A)** Amplification of the *PDL1/JAK2* locus at *9p24* in a primary mediastinal B-cell lymphoma (PMBCL); note multiple green FISH signals corresponding to the locus of interest compared to only 2 red centromere 9 signals/cell. **(B)** Rearrangement of the *CIITA* locus at *16p13* a PMBCL; note fused green and red signals corresponding to the non-rearranged wild-type allele and free green and red signals corresponding to the rearranged allele.

An important feature of cHL [and primary mediastinal B-cell lymphoma (PMBCL)] is gain of chromosome *9p24* (Figure 2A), which leads to an overexpression of PDL1 (25, 26) that can also be shown *in situ* (27, 28), and seems to be of probable prognostic importance in patients treated with standard treatment regimens (25) and offers the opportunity to be specifically targeted, resulting in unprecedented response rates in otherwise hopeless cases of multiple-relapsing cHL (29). Other genes in this region encompass *JAK2, PDL2*, and *JMJD2C*, the upregulation of all of which seems to be vital for HRS cells (30), explaining why blocking PD1 might be more effective than blocking PDL1 in cHL as the first might prevent the tumor cells also from relying on PDL2 as a substitute of blocked PDL1 (31).

Other immune escape mechanisms in cHL (and PMBCL) are deactivating translocations of *CIITA* (Figure 2B), the transactivator gene of MHC class II, that can be found in a subset of cHL (32), and downregulation of MHC class II that is reported to be also an adverse prognostic factor in affected individuals (33). The same applies to MHC class I (34), although this study could not confirm the impact of MHC class II as a prognostic factor. Regarding MHC class I, mutations in the *B2M* gene, which is important for MHC class I composition and function, are among the commonest in cHL and have been shown to be a predictor of inferior outcome independently of the *9p24* status (34).

CD58, also known as lymphocyte function-associated antigen 3, is a glycosylated surface molecule on both B- and T-cells, which provides a stimulatory signal for T cells *via* the CD2 receptor. The function of CD58 in cHL is two-faced: on the one hand, it is necessary for HRS cells to communicate with CD4-positive T-cells (35), on the other hand, loss of CD58 expression due to mutations can facilitate immune escape, especially in advanced disease, when HRS cells become less dependent on the surrounding microenvironment (36, 37). HLA-G, a non-classical HLA molecule, plays a similar role in cHL and modulates the microenvironment to foster immunotolerance. HLA-G expression has been demonstrated both on HRS cells and the microenvironment, with high HLA-G expression on HRS cells and, conversely, low expression in the microenvironment correlating with a better outcome in one study (38).

Epstein–Barr virus (EBV) infection of HRS cells is a common feature in 30% of cHL in the Western world and >90%—especially in pediatric cases—in Central America (39). EBV infection is clonal and, thus, an early event in cHL. It immortalizes B-cells by rescuing them from apoptosis (40). EBV shows latency II state in HRS cells, with expression of the EBV-encoded genes EBV nuclear antigen 1 (EBNA1), latent membrane protein 1 (LMP1), and LMP2a. In EBV-negative cHL, the oncogenic impact of EBV seems to be substituted by mutations of genes related to the NF-κB pathway (e.g., *C-REL*) as well as several receptor tyrosine kinases (41). EBV can also upregulate PDL1 expression (42). This is primarily mediated by LMP1. LMP1 activates both the JAK/STAT pathway directly *via* JAK3 as well as activated protein 1 (AP1) *via* the microtubule-associated protein kinase (MAPK) pathway, both of which promote *PDL1* gene expression (42). Interestingly, while frequencies of *9p24* gains and amplifications are similar in EBV-positive and EBV-negative cHL, PDL1 expression is mostly and more selectively upregulated in EBV-positive cHL (25). EBNA1 and LMPs also directly interact with immune cells helping to create an immunosuppressive environment with enhanced amounts of regulatory T-cells (43).

Finally, HRS cells secrete a plethora of immunosuppressive soluble mediators, which is beyond the scope of this review (44, 45).

## Various Mechanisms of Immunomodulation in Lymphomas—A Closer Look

In the second part of this review and after having focused on one specific lymphoma subtype, which is the prototype for immunomodulative cancer, we will have a closer look at the various mechanisms touched in the previous sections, namely, PD1/PDL1, CTLA4/B7, HLA-G, CD58 and B2M, CD70, and CD27 as well as EBV. Beside a discussion on how these pathways exert their function and by which types of lymphomas they are used, we will also focus on interactions between them and show their synergistic and/or complementary mode of action.

### PD1/PDL1—The Best Studied and Most Frequently Therapeutically Used Pathway of Immune Evasion

PD1 and its ligand PDL1 have already been discovered in the early and late 90s, respectively (46, 47). A second ligand of PD1, PDL2, the expression of which is more restricted than that of PDL1, has been identified as well (48). These molecules are important tools to control T-cell activity and proliferation, and can both inhibit T-cells as well as stimulate immunosuppressive regulatory T-cells (49, 50). Another recently discovered ability is the effect of PDL1 on TAM briefly touched in the section on cHL. Gordon et al. recently showed that PDL1 blockade increases the phagocytic capability of TAM in rodent models and leads to increased survival and tumor control (19). This is an interesting and potentially also clinically relevant finding considering the bad prognostic effect of high numbers of TAM in cHL (51, 52), which might thus be counterbalanced by PDL1 inhibition. In contrast to CTLA4, which is discussed in the next paragraph, PD1 and its ligands exert their function in the peripheral tissue and thus do not lead to a systemic affection of the immune system, which has been nicely shown in several animal models (53, 54). The cytoplasmic tail of PD1 contains an immunoreceptor tyrosine-based switch motif (ITSM) and an immunoreceptor tyrosine-based inhibitory motif (ITIM), of which the ITSM is essential for the transmission of inhibitory signals [reviewed in Ref. (55)]. Upon T-cell receptor (TCR) stimulation and ligation with either PDL1 or PDL2, the ITSM and ITIM undergo phosphorylation, leading to the recruitment of the phosphatases SHP-1 and SHP-2, which in turn lead to dephosphorylation (inactivation) of the crucial T-cell signaling molecules ZAP70 and CD3ζ, and, in addition, of the phosphatidylinositol 3-kinase, which interrupts AKT and ERK signaling; even more, upon PD1 engagement by PDL1, protein tyrosine kinase-θ, which is necessary for the activation of the transcription factors NFκB and AP1, is attenuated and the negative regulator of T-cell activation, the E3 ubiquitin ligase CBL-b is upregulated (56–58). As a net effect, TCR-mediated activation and T-cell proliferation are impeded.

PD1/PDL1 expression in lymphomas (Figures 1A,B) has been investigated by a variety of studies with mostly consistent results (27, 28); it can be demonstrated in up to a third of DLBCL, mainly of the activated B-cell type (59), and in PMBCL, in other lymphoma entities it is expressed in only a low percentage of cases (27). Interestingly, in chronic lymphocytic B-cell leukemia (CLL), PDL1 expression has been described in the proliferation centers (60). PDL1 expression is observed both in the tumor microenvironment (particularly in tumor-infiltrating macrophages) and in lymphoma cells, while PD1 is primarily expressed in T-cells of the microenvironment. In T-cell- and histiocyte-rich B-cell lymphomas, PDL1 expression is seen in both T-cells and histiocytes, while the tumor cells themselves are negative for PDL1 (27). Importantly, in extranodal natural killer (NK)- and T-cell lymphoma of the nasal type, which is known to have an aggressive and mostly fatal course, PDL1 is substantially upregulated due to EBV infection of the tumor cells, and PD1 blockade has been shown to be very effective in otherwise hopeless relapse cases in a small case series (61).

As mentioned above, the genetic mechanism of PDL1 overexpression has been first elucidated in cHL consisting of alterations in chromosome *9p24.1*. Similar alterations have been found in PMBCL (62) and DLBCL (63) as well as lymphomas of immune-privileged sites such as the central nervous system and the testis (64). In addition to gene gains, PDL1 expression is inducible by LMP1 of EBV *via* activation of STAT- and AP1-mediated pathways. As to be expected, other causes of STAT activation also enhance PDL1 expression as seen in anaplastic lymphoma kinase-positive anaplastic large cell lymphomas (65) or in instances with active cytokine signaling (66). Another mechanism of enhancing PDL1 expression was just recently reported by Kataoka et al., who demonstrated the presence of disruption of the 3′-untranslated region (UTR) of the *PDL1* gene leading to a marked increase of PDL1 that is stabilized by truncation of the 3′-UTR (67). Finally, at least in DLBCL, translocations of *IGH, PIM1*, and *TP63* with the *PDL1* locus that lead to latter’s overexpression have been described, too (63).

As in solid tumors, the direct applicability of PD1/PDL1 expression to predict therapy responsiveness and prognosis remains to be fully elucidated. Xing et al. could show that PDL1 expression in DLBCL treated with standard R-CHOP treatment is associated with a better overall survival rate, yet not with remission after first therapy, relapse- or progression-free survival (68). Several studies with small patient cohorts suggest that best responses are seen in lymphomas harboring *9p24* alterations such as lymphomas of immunoprivileged sites (69). In PMBCL, high PDL1 expression and low MUM1p expression is correlated with a better outcome than *vice versa* expression of these two proteins (70). A study on refractory lymphomas revealed that there is a discrepancy between PDL1 expression and amplification of the *PDL1* locus, supporting the hypothesis that other mechanisms—next to gene amplifications—are involved in upregulation of PDL1 expression (71). It has also become evident that in several lymphoma types such as follicular lymphoma and CLL, adding PDL1 blockers to conventional therapy regimens shows a benefit in comparison to only very limited treatment response if given as single agents (72). For comprehensive overviews of ongoing and already finished clinical trials, we refer to several recent excellent clinically centered reviews as well as the contributions of Proff. Renner and Stenner in this issue.

### CTLA4—A Key Player Seemingly Not Only in T-Cell Lymphomas

CTLA4 belongs to the superfamily of immunoglobulins (73). It is generally expressed in T-cells, and regulatory T-cells are constitutively positive (74). It shares its ligands B7-1 (CD80) and B7-2 (CD86) together with CD28, which has a function opposite to CTLA4 as it is a stimulator of TCR signaling (75). CTLA4’s affinity and avidity to these ligands is greater than that of CD28 due to its bivalent binding to the B7 molecules (76). The main function of CTLA4 is T-cell inactivation, which is exerted by two different mechanisms: it competitively binds its ligands B7-1 and B7-2 leading to a reduced stimulatory signaling of CD28; furthermore, *via* its cytoplasmic tail, CTLA4 can inhibit various intracellular signaling pathways in T-cells such as NF-κB, AP1, and nuclear factor of activated T-cells (77), it can impede the cell cycle (78) and inactivate MAPK, extracellular signal-regulated kinase-1 (ERK) and c-Jun NH2 terminal kinase signaling, and thus impair interleukin 2 production (79). In contrast to PD1/PDL1, which exert their function in the periphery, CTLA4 is acting rather early in the time course of the immune response as it is involved in priming T-cells in primary lymphoid organs (80).

CTLA4 expression is noted in a variety of T-cell lymphomas, namely, peripheral T-cell lymphomas and mycosis fungoides/Sézary syndrome. Besides inducing T-cell anergy and, thus, fostering immune escape, CTLA4 has also a direct oncogenic effect: a fusion of the two opponents CTLA4 and CD28 has recently been described in a variety of T-cell lymphomas and proposed to be a major driver of lymphoma development (81). The fusion protein consisting of the extracellular and transmembrane domains of CTLA4 and the cytosolic signaling domain of CD28 showed increased activation of intracellular MAPK and ERK signaling in cell culture experiments, confirming observations of earlier studies (82). Herrmann et al. reported CTLA4 expression in B-cell lymphomas, primarily in DLBCL (83). These lymphomas were shown to be able to exert their immunosuppressive function by binding of B7.1 and thus reducing CD28 activation on tumor-infiltrating/immunosurveillance T-cells; furthermore—as in T-cell lymphomas—CTLA4 can enhance proliferation *via* the STAT3 pathway, which is an important driver also in B-cell lymphomas (84). So far, CTLA4 inhibition is not commonly used in lymphoma therapy. In cHL, CTLA4 blockade has been tested in transplanted patients (85) and in combination with brentuximab, the latter still being an ongoing trial (86).

### HLA-G—The Unknown Member of the HLA Family

**Figure 3:**
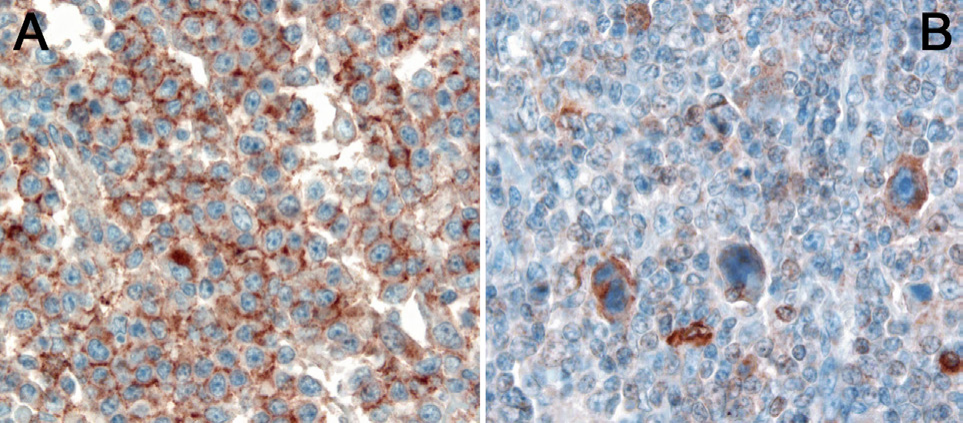
**(A)** Expression of HLA-G in a diffuse large B-cell lymphoma. **(B)** Expression of HLA-G by Hodgkin- and Reed–Sternberg cells of classical Hodgkin lymphoma.

HLA-G is a non-classical MHC class I molecule and besides the classical function of HLA proteins—presenting protein fragments on the cell surface—it exerts its function mainly by immunomodulation (87). In contrast to the classical HLA molecules, the non-classical HLA are highly conserved molecules with only few alleles. Immunomodulation by HLA-G occurs *via* a plethora of ways as it can interact with different receptors found on T-cells, B-cells, macrophages, dendritic cells, and NK cells (88). It interferes with proliferation and cytotoxicity as well as promotes apoptosis. Furthermore, it also inhibits chemotaxis by downregulating several chemokine surface receptors (89), hampers the function of neutrophils (90), and reduces neoangiogenesis (91). HLA-G expression has been investigated in a variety of cancers and is correlated with worse overall survival or increased risk of tumor progression and metastases in most studies (88). In lymphomas, HLA-G has been explored in only few studies so far and the results regarding the predictive role of HLA-G expression are still equivocal (92). As alluded to above, HLA-G expression has been demonstrated in cHL (Figures 3A,B) and its high expression in the tumor microenvironment has been correlated with an inferior response rate (38). Bielska et al. demonstrated that *HLA-G* polymorphisms, which have a direct impact on the expression of HLA-G RNA, differ between different prognostic groups of DLBCL (93), and similar findings were reported in CLL patients (94). Both studies showed independently that especially the 14 base pair deletion polymorphism (rs66554220) in the 3′ UTR of *HLA-G* has an adverse prognostic impact.

### CD58 and B2M—Important Prerequisites for Immunosurveillance

Both CD58 and B2M are important for the correct assembly of MHC class I molecules (95) and alterations thereof are another immune escape mechanism of tumors (96). Inactivating mutations of *CD58* have been initially described in approximately one sixth of DLBCL with no preference for either cell of origin subtype (97). They are as frequent as mutations of *B2M*; in our study on 76 DLBCL in immunocompetent patients, the mutational frequency of *B2M* was 16% (98). Interestingly, loss of CD58 cell surface expression is more commonly observed than assumed from its mutational frequency and many DLBCL show a concomitant loss of HLA class I and CD58. As loss of HLA class I alone might increase susceptibility to lysis by NK cells (99), the concomitant loss of CD58, which is a CD2 ligand, might act in a counterbalancing way. The reduced cytolysis of DLBCL cells lacking CD58 expression has been confirmed in cell culture experiments (97). *CD58* mutations have also been described in a small percentage of peripheral T-cell lymphomas along with *B2M* mutations (100). Mutations of *CD58* and *B2M* are thought to be a main reason for non-responsiveness to immune checkpoint inhibition (101). Cao et al. showed that both mutations and copy number losses of *CD58* and *TP53* genes are independent unfavorable prognostic factors in DLBCL (102). This is the first study attributing such a high impact to *CD58* mutations.

*B2M* mutational rates vary in specific subtypes of DLBCL: in DLBCL of the testis and the central nervous system, i.e., DLBCL arising in immunoprivileged sites, *B2M* mutations have been reported to be frequent (103), while in our study on posttransplant DLBCL, no *B2M* mutations were detected (104). From this finding, we concluded that *B2M* mutations do not provide an additional advantage in the state of immunosuppression as there is, for obvious reasons, no genetic pressure for immune escape on the tumor cells.

### The CD70–CD27 Axis: Another Key to T-Cell Control

**Figure 4:**
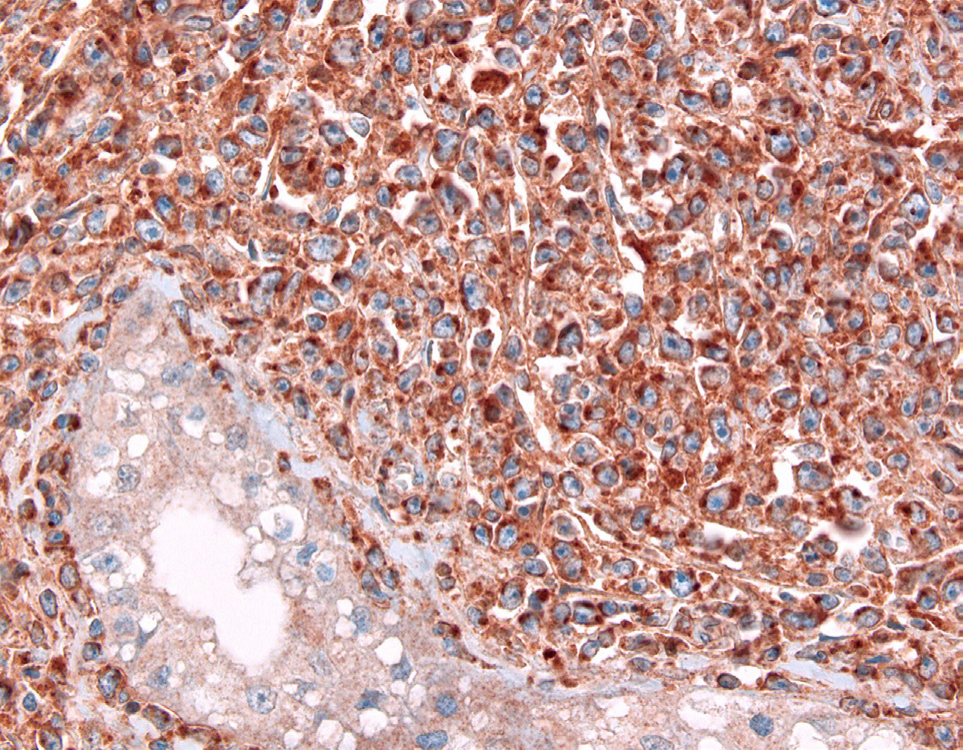
Expression of CD70 in a testicular diffuse large B-cell lymphoma; note a negative seminiferous canaliculus.

CD27 belongs to the tumor necrosis factor family; it is involved in the activation of both innate and adaptive immunity. It is expressed in thymocytes and naïve T-cells as well as activated T-cells (105), memory B-cells (106), and NK cells in the bone marrow but not in circulating NK cells (107). CD27 has a unique ligand, CD70, which has become a focus of potential therapeutic interaction. A plethora of different tumor entities including many lymphomas (Figure 4) have been shown to express CD70 (108, 109), whereas CD27 expression is primarily restricted to hematopoietic tumors (108). Tumors use the CD70–CD27 axis in order to manipulate T-cells in an immunosuppressive manner by increasing the proportion of inhibitory regulatory FoxP3^+^ T-cells (110), induction of T-cell apoptosis (111), and skewing T-cells toward anergy and exhaustion (112).

First studies using monoclonal antibodies directed against CD70 have been tested with rather low response rates [complete remission in 1/19 lymphoma patients (113)]. Currently, several trials of combining anti-CD70 therapy and chemotherapy and radiotherapy are ongoing. The rationale behind this approach is that by activating the immunosurveillance of the microenvironment by CD70 blockade, the effect of conventional chemotherapy and radiotherapy is increased (114).

### EBV—The Classical Model of Oncogenicity and Immune Escape

Epstein–Barr virus’s role in lymphomagenesis was first discovered in Burkitt lymphoma (BL). While the *MYC* translocation is important for upholding the proliferative activity of BL, the main effect of EBV is thought to be effectively preventing c-myc-induced apoptosis (115). EBV-infected non-neoplastic memory B-cells express only one EBV-specific protein (EBNA1)—known as “latency type 1”—in order to avoid recognition by the immune system, and these cells provide the life-long reservoir of EBV in humans. This latency type 1 is sustained in many B-cell lymphomas including BL, DLBCL and terminally differentiated B-cell lymphomas, while in cHL and many NK- and T-cell lymphomas, virus-infected tumor cells express to a certain extent LMP1 and LMP2A&B (without EBNA2), known as latency type 2, and in lymphomas of immunosuppressed individuals EBNA2-3C are expressed along with LMPs, referred to as latency type 3 (116). Importantly, latency type 2 is an intriguing therapeutic target for PD1/PDL1-blocking agents as exemplified in cHL and NK/T-cell lymphomas (29, 61), while the latter latency type 3 would be recognizable by a functional immune system and is tolerated due to the concomitant immunosuppression in affected individuals as exemplified by recurrent tumor control in seldom instances, in which the respective immunosuppression can be restored (117, 118) (see also: expansion of decreased T helper 1 and CD8^+^ T cell subsets associates with regression of lymphoproliferative disorders developed during methotrexate treatment. Saito et al., published in the same journal issue). Even more, EBV relatedness in several of the above listed instances may even stand for distinct diseases, as it has been shown for DLBCL and PTLD (104) and recently also for plasmablastic lymphoma (119) that EBV-positive and EBV-negative tumors have both different pathogenesis as well as different prognosis. EBV exerts effects on the tumor cells related to proliferation and preventing apoptosis, and on the microenvironment, particularly on the host’s immune system. In the setting of human immunodeficiency virus (HIV) infection, a marked increase of EBV-related lymphomas has been initially observed (120). With the introduction of highly active antiretroviral therapy (HAART), the incidence of HIV-related lymphomas has considerably changed: while there was a steep decline of EBV-associated lymphomas of the CNS and DLBCL, cHL incidence has risen, and the incidence of BL has remained stable (121). This shows that the risk to develop certain types of lymphoma is related to the function of the immune system. While several subtypes thrive in severe immune suppression (EBV-related DLBCL in general), cHL is dependent on an at least partially functioning immune system due to HRS cell interaction with the microenvironment, particularly their dependence on CD4^+^ T-cell signaling (122), and thus their restoration by HAART “paradoxically” promotes cHL development. In BL, it is postulated that the expansion of the germinal center reaction and the pronounced activation of polyclonal B-cells seen in the early stages of HIV—induced by several viral proteins (123)—increases the amount of EBV-infected B-cells with *MYC* translocations (115). This reservoir of translocated and virus-infected B-cells, already “replenished” at the very beginning of HIV infection, increases the risk of BL outgrowths, which is independent of future control over HIV.

Apart from improving T-cell function and numbers, a key to treatment of EBV-related lymphoma is modulation of the ubiquitin–proteasome system. This vital cell component is used by EBV in several ways: it is inhibited by the virus to foster immune evasion (124); furthermore, it is used for modulation of cell cycle checkpoint proteins such as proto-oncogene serine/threonine protein kinase 1 (PIM1) (125) or tumor suppressors such as p16 and retinoblastoma protein (pRb) (126); finally, it is involved in inhibition of apoptosis by fostering degradation of p53 and BCL6. The proof of concept of inhibiting the ubiquitin-proteasome system has been delivered in several EBV-associated malignancies (both carcinomas and lymphomas); however, larger clinical trials for testing this approach in the clinical setting are still required (127). In plasmablastic lymphoma, which is EBV-associated in the vast majority of cases (128), bortezomib treatment has already shown considerable improvement of treatment response and survival rates in small cohorts (129).

### Hematological Side Effects of Immunomodulative Therapies

Adverse events (AE)/side effects of immune checkpoint inhibition drugs are reported to be rarer than those of classical chemotherapy agents (130). In contrast to the well understood genesis of pathologic changes in peripheral organs, which can mainly be explained by a graft-versus-host-like pathophysiology, the underlying mechanisms for hematological side effects of checkpoint inhibitors are not yet fully understood. Hematological AE in general seem to be more common in lymphoma patients than in patients treated for solid tumors (131). They manifest as isolated neutropenia, thrombocytopenia, or anemia, in some cases as pancytopenia, which may all have in common decreased auto-tolerance mechanisms under immunomodulation (132). Furthermore, development of hemophilia A in patients treated with anti-CTLA4 antibodies has been described (133, 134). In one study on DLBCL patients, a condition referred to as myelodysplastic syndrome (MDS) occurred in a single patient and was listed among the AE (131). However, in our point of view, it is difficult to attribute a MDS to immune checkpoint inhibition as several potential other causes should be considered (e.g., therapy-associated myeloid neoplasm after several previous chemotherapy courses!) and the mechanism how immune checkpoint inhibitors entice MDS-related mutations remains completely unclear. Though the pathophysiology of hematological AE seen in the context of immunomodulative therapies is not fully elucidated yet, it is highly likely that they develop in an autoimmune disease-like manner. In AE suspect instances, it is vital to rule out other potential causes of cytopenias such as lymphomatous bone marrow involvement, substrate deficiencies or toxicities of former (chemo-)therapies including evolving therapy-associated myeloid neoplasms, concomitant treatment with myelotoxic medications, e.g., certain NSAR, mycophenolate, or mTOR inhibitors (135). Interestingly, occurrence or worsening of graft-versus-host disease (GvHD) in previously transplanted individuals, whom immune checkpoint inhibition was given, has been reported as an AE in some studies, while others reported a reduced incidence (132). Importantly, in an experimental setting, PDL1 inhibition reduced GvHD without hampering the graft versus lymphoma effect in mice (136).

## Conclusion

**Figure 5:**
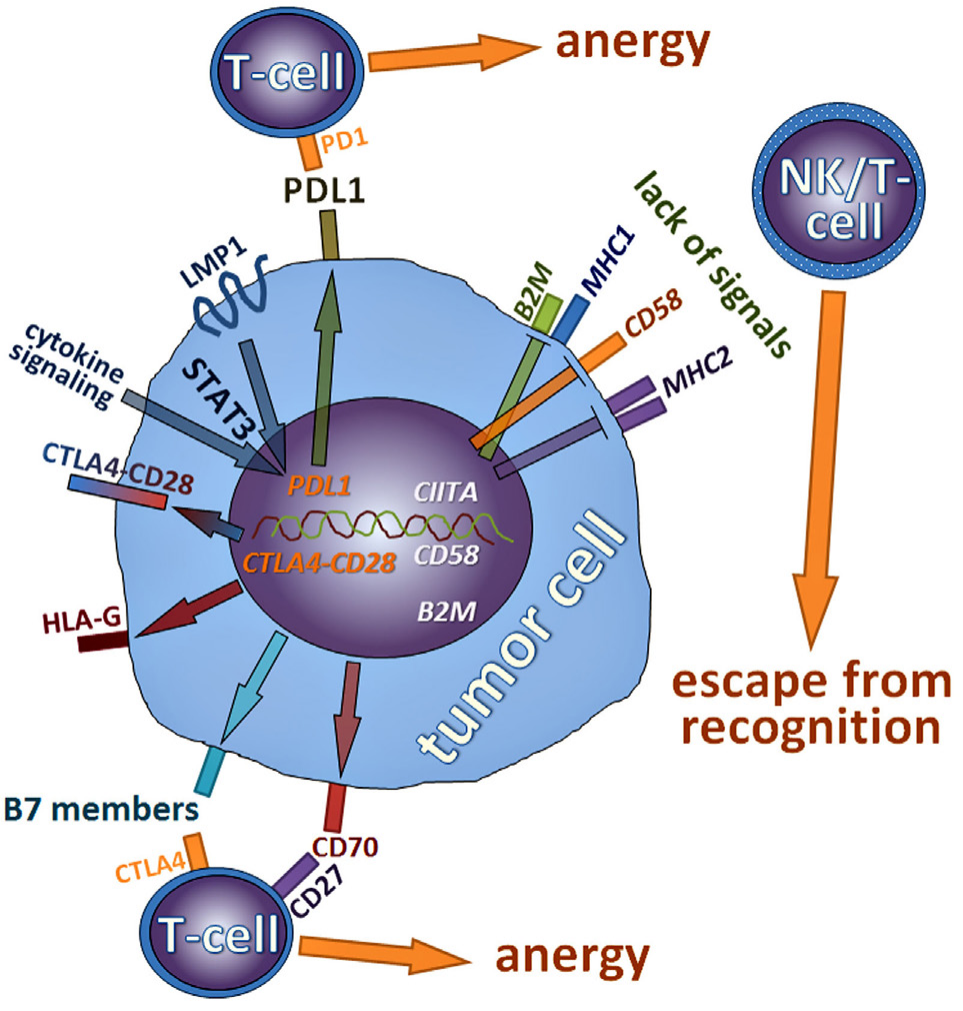
Schematic summary of mechanisms discussed in this review lymphoma cells employ to influence or circumvent the immune system; molecules that are rather repressed are in italics; genes inactivated by mutations are in white color, while those activated are in orange.

In this review, we have summarized mechanisms lymphoma cells employ to influence or circumvent the immune system (Figure 5). We have shown that many mutations and pathway alterations discovered in cHL—the pathognomonic example for a lymphoma interfering with the immune system—can also be found in other types of lymphomas and that these alterations, to which many lymphomas are oncogenically addicted, can be specifically targeted. Indeed, it has become evident that manipulating the immune system taints to be an advantageous management strategy for many tumors including lymphomas. Thorough research has elucidated several mechanisms how this is achieved, it has also become clear that both tumor cells and microenvironmental compounds should be considered and modulated in a proper manner. These findings have led to a plethora of new potential treatment options, which have already proven to be beneficiary for patients.

However, it has also become evident that there is no uniform treatment response, highlighting the need for individualized analysis of patients’ tumors and the corresponding individual immunological/immunogenetic background in order to decipher on the one hand the specific pathways used by the tumor to hamper the hosts’ immune system and the potential responsiveness of the latter. It has also become evident that immunotherapy can and probably should be synthetically combined with the other pillars of cancer therapy—surgery, chemotherapy, and radiotherapy—as this can markedly improve the impact of each therapy approach.

## Author Contributions

Both AT and TM conceived and wrote the manuscript.

## Conflict of Interest Statement

The authors declare that the research was conducted in the absence of any commercial or financial relationships that could be construed as a potential conflict of interest.
